# Natural Products and Pharmacological Properties of Symbiotic Bacillota (Firmicutes) of Marine Macroalgae

**DOI:** 10.3390/md21110569

**Published:** 2023-10-30

**Authors:** Uche M. Chukwudulue, Natalia Barger, Michael Dubovis, Tal Luzzatto Knaan

**Affiliations:** Department of Marine Biology, The Charney School of Marine Sciences, University of Haifa, Mount Carmel, Haifa 103301, Israel; chukwudulueuche@gmail.com (U.M.C.); nbarger@univ.haifa.ac.il (N.B.); mdubovis@univ.haifa.ac.il (M.D.)

**Keywords:** seaweeds, macroalgae, Firmicutes, Bacillota, natural products, secondary metabolites, biological properties, pharmacological activities

## Abstract

The shift from the terrestrial to the marine environment to discover natural products has given rise to novel bioactive compounds, some of which have been approved for human medicine. However, the ocean, which makes up nearly three-quarters of the Earth’s surface, contains macro- and microorganisms whose natural products are yet to be explored. Among these underexplored marine organisms are macroalgae and their symbiotic microbes, such as Bacillota, a phylum of mostly Gram-positive bacteria previously known as Firmicutes. Macroalgae-associated Bacillota often produce chemical compounds that protect them and their hosts from competitive and harmful rivals. Here, we summarised the natural products made by macroalgae-associated Bacillota and their pharmacological properties. We discovered that these Bacillota are efficient producers of novel biologically active molecules. However, only a few macroalgae had been investigated for chemical constituents of their Bacillota: nine brown, five red and one green algae. Thus, Bacillota, especially from the marine habitat, should be investigated for potential pharmaceutical leads. Moreover, additional diverse biological assays for the isolated molecules of macroalgae Bacillota should be implemented to expand their bioactivity profiles, as only antibacterial properties were tested for most compounds.

## 1. Introduction

Natural products are molecules produced by organisms that have played a vital role in drug discovery [1]. Generally, the terrestrial environment and its organisms are well-studied for bioactive compounds, and this is due to the relative ease of their sampling [2] and culturing in the laboratory [3]. However, the largest ecosystem, the ocean [4], abundantly endowed with chemical and biological resources [5], is less explored [6,7]. The challenging conditions of the marine environment, for example, high salt concentration, low temperature, high pressure, low nutrients, and varying light intensities [8], spur marine organisms to synthesise uncommon chemical compounds that help them adapt to their environment [9]. Recently, researchers have been moving away from terrestrial to the marine ecosystem for unique therapeutic molecules with high pharmaceutical and biotechnological potentials [10]. However, many more studies are needed to cover the ocean’s vast biodiversity for this purpose.

So far, about twenty marine natural products (MNPs) are approved as pharmaceutical drugs [11], and twelve of them are specifically for cancer treatments [12]. Despite the vast number of microbes in the marine ecosystem, the number of microbial-derived marine drugs is limited [11]. Investigating more marine microbes, especially symbionts, for drug-like compounds is necessary, since the actual producers of many natural products are microorganisms inhabiting their hosts [13]. A notable example is tetrodotoxin, initially isolated from pufferfish and octopus but later discovered as a secondary metabolite of some symbiotic bacteria [14,15]. Another example is Taxol, previously obtained from a terrestrial plant, the Pacific yew [16], which was found afterwards to be a product of an endophytic fungus of the plant. Another reason to pursue symbiotic marine microorganisms is their ability to produce a greater variety of novel secondary metabolites than their free-living counterparts [17].

Marine macroalgae (seaweeds) are common hosts of diverse bacterial species [18], and algae–bacterial association is usually host-specific [19,20,21]. Host specificity means that some algae accommodate more microbial species than others [22], and similar microbial species colonise the same algae found in different ecological niches [19]. This algal holobiont, which refers to the host algae and its associated microorganisms, is influenced by the type of nutrients the host provides [23]. In addition to the metabolic duties performed by the bacterial symbionts for their hosts, they also protect them from harmful organisms and substances by producing antimicrobial and antifouling agents [18,24,25]. This advantage is due to the chemical interactions between the host and its associates [26] caused by the quest for survival in a competitive environment [27].

Alongside Actinomycetota and Pseudomonadota, Bacillota has been identified as the bacterial phylum most associative to marine organisms, including algae [28,29,30]. It is a ubiquitous phylum of bacteria found in both terrestrial and aquatic environments. Species of Bacillota are found abundantly in different regions of the human body [31,32,33], animals [34], soil [35,36], plant stems [37] and rhizospheres [38,39]. In the aquatic habitat, they can exist in water [40] or sediment [41], or associated with higher marine organisms [24,42,43]. This phylum of bacteria comprises mainly Gram-positive species exhibiting diverse phenotypic forms, most thriving at neutral pH [44]. They form endospores that enable them to adapt to many ecological conditions and are easily cultured in laboratories [45].

Bacillota are among the large genome-sized bacteria that use over 9% of their genomes to encode novel biosynthetic gene clusters (BGCs) that produce new bioactive compounds [46,47]. These bioactive molecules belong to different chemical classes, such as terpenes, polyketides (PKs), non-ribosomal peptides (NRPs), lasso peptides, bacteriocins, thiopeptides, ectoine, and melanin [28]. Though Bacillota dedicate a significant portion of their genome to producing bioactive compounds, most approved microbial drug products, especially antibiotics, are from a different bacterial phylum, Actinomycetota [48,49]. So far, Bacillota has only a few products on the market.

In human medicine, a deadly toxin (botulinum toxin type A) produced by *Clostridium botulinum*, commonly found in soil, has been developed into a vital drug (Botox^®^) [50]. Botox^®^ is widely used to treat health issues ranging from strabismus to spasticity [51,52]. Studies have also tested its use in treating bruxism [53] and cosmetic procedures [54], and its anticancer properties [55,56]. Another FDA-approved product of Bacillota is polymyxin B, a chemical compound first obtained from a soil bacterium, *Paenibacillus polymyxa* [57]. This antibiotic is the last-resort treatment for multidrug-resistant Gram-negative bacterial infections [58]. Other formulated products from Bacillota are nisin (E 234), an antibiotic food additive isolated from a subspecies of *Lactobacillus lactis* [59,60], and two enzymes (protease and carbohydrase) from *Bacillus subtilis* or *Bacillus amyloliquefaciens*, considered to be GRAS (generally recognised as safe) by the FDA [61].

Some commercially available plant biofungicides from Bacillota in the agricultural industry include SERENADE^®^, Double Nickel 55^TM^ and other products. These products are formulated from *B. velezensis*, a member of the operational group *Bacillus amyloliquefaciens* (OG*Ba*) made up of *B. amyloliquefaciens*, *B. siamensis*, *B. velezensis* and *B. nakamurai* [62]. In addition, about 50% of approved plant bacterial biocontrol formulations used in different countries are *Bacillus* species products [63]. Examples include BioPro^®^, Rhiso Plus^®^, Biosubtilin, Botrybel^®^, and NacillusPro™ [64] and also the animal probiotic Ecobiol Plus^®^ from *B. amyloliquefaciens*, commercially available in Europe for pigs and chickens, with potential use in aquaculture [65].

Notwithstanding the low popularity of natural products of Bacillota in the clinical realm, some of the biological properties of macroalgae Bacillota, especially the antimicrobial effects, have previously been captured in scientific reviews [66,67]. However, to our knowledge, no summary of the natural products and corresponding biological effects of Bacillota associated with marine macroalgae is available. Therefore, we summarise the chemical compounds and pharmacological properties of symbiotic Bacillota of marine macroalgae so far investigated. This survey will guide interested researchers through the existing information regarding natural products and bioactivities of macroalgae symbiotic Bacillota, aiming to spark motivation to expand the research in the field.

## 2. Aquatic Bacillota

A study by da Silva et al., 2013 identified at least a strain of Bacillota in every analysed sample of sea sediment collected from different depths of the South Atlantic Ocean, in contrast to other phyla [68], corroborating the easy cultivation of marine Bacillota in the laboratory [69]. Also, while assessing the microbial symbionts of sponges and a soft coral obtained from the Red Sea, Refs. [13,70] found that Bacillota was the most-encountered bacteria phylum, usually synonymous with bioactive secondary metabolites. For instance, some marine *Bacillus* species produced many novel natural products, ranging from macrolides [71,72,73] to fatty acids [74]. A specific example is *Bacillus silvesteris* from a marine crab, which synthesised two unknown cyclodepsipeptides with very high cytotoxic effects (GI_50s_: 0.001–0.01 ng/mL) against human cancer cell lines [75]. Unfortunately, with these exciting numbers of novel molecules associated with marine Bacillota, only a few studies covered Bacillota from macroalgae; more studies were on Bacillota of corals and sponges.

## 3. Marine Macroalgae, a Good Source of Bioactive Bacillota 

Consistently, marine macroalgae have produced various remarkable molecules [2,26]. Between 1960 and 2012, more than three thousand natural products were identified in different algae types, and they are still receiving attention for their novel bioactive compounds [76]. Some examples of algae chemical compounds include two potential anticancer agents, lophocladines B from a red alga [77], dieckol from a brown alga [78] and Griffithsin from a red alga, currently in phase I clinical trial for HIV prevention [11,79]. However, algae’s commercial and ecological applications are linked to the chemical communications between them and their associated microbes [18,80]. To ascertain this assumption, scientists are investigating algae microbial symbionts for their natural products [19,81,82,83]. It appears that Bacillota play more beneficial than pathogenic functions in algae [22,81], by producing chemical compounds that protect algae from fouling and colonising pathogenic microbes. Subsequently, we will delve into these chemical arsenals produced by the algae Bacillota to defend themselves and their hosts. 

## 4. Secondary Metabolites of Marine Macroalgae Bacillota and Their Biosynthetic Gene Clusters

Several chemical compounds have been obtained from marine macroalgae-associated Bacillota in laboratories. However, the type of compounds generated by any microorganism in a conventional laboratory can be influenced by the properties of the growth medium [13]. Different classes of compounds would emerge by varying the composition or condition of the culture media. For instance, growing a symbiotic bacterium in a static culture or one consistently shaken at a particular speed affects the types of chemical compounds the bacterium will produce [82]. This is a well-known natural-product drug-discovery approach called the one strain many compounds (OSMAC) approach. To capture the effects of the OSMAC strategy, we included in Table 1 various culture media in which the algae Bacillota were grown and the resulting chemical compounds. From Table 1, we can see that researchers increased the chance of recovering a diverse range of bacteria species from algae samples by employing multiple culture media at the isolation step. After this step, they selected the specific media most suitable for the growth and purification of their target bacterial species. Finally, they cultivated each pure bacterium strain in a medium that promotes the production of bioactive chemical compounds.

**Figure 1:**
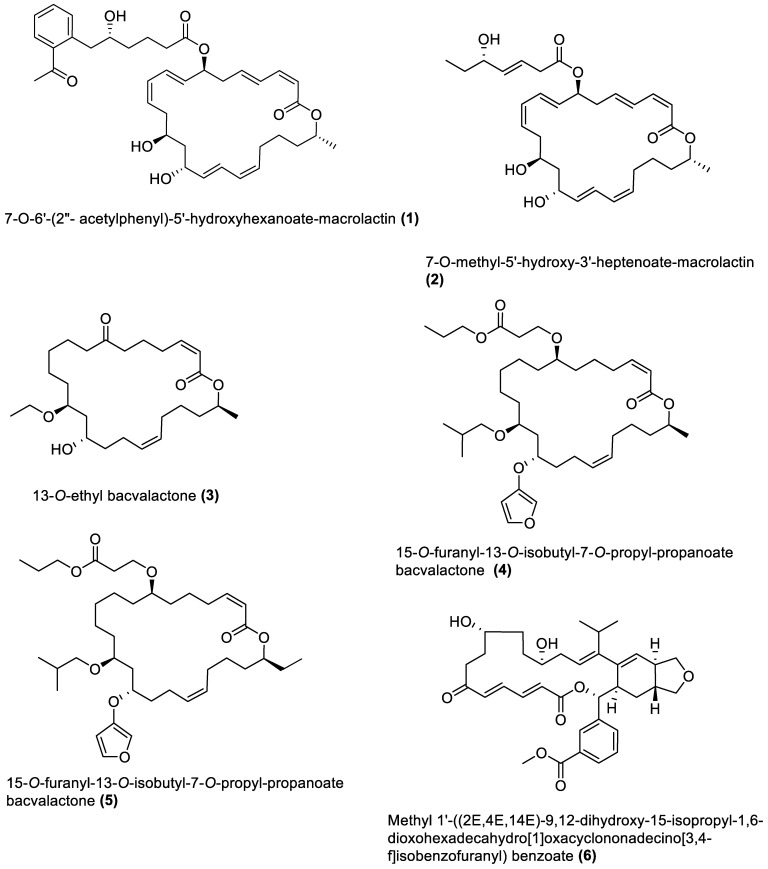

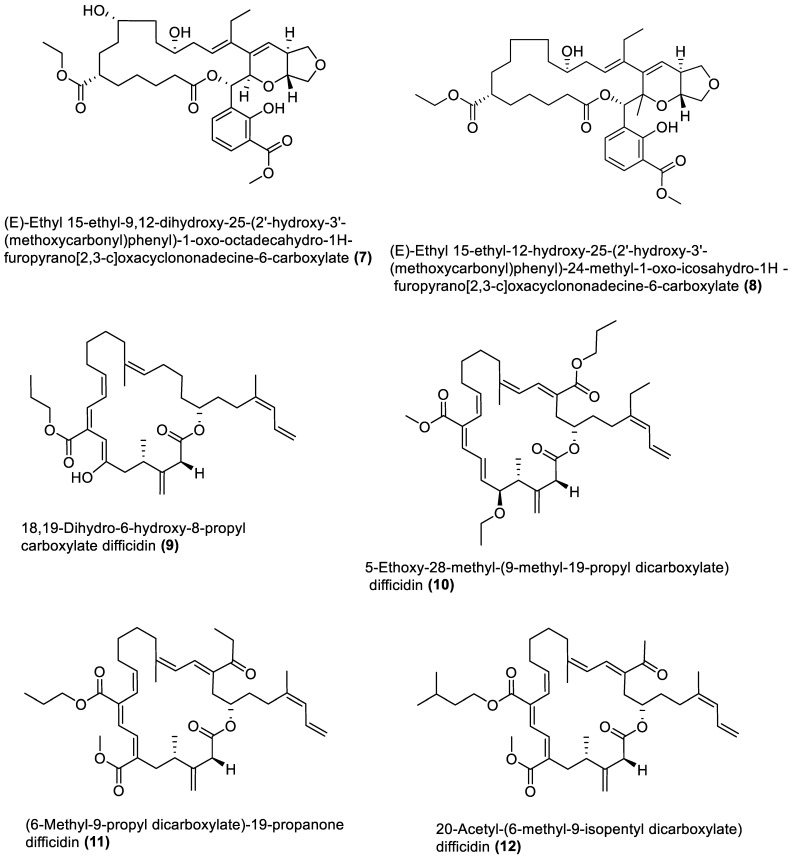

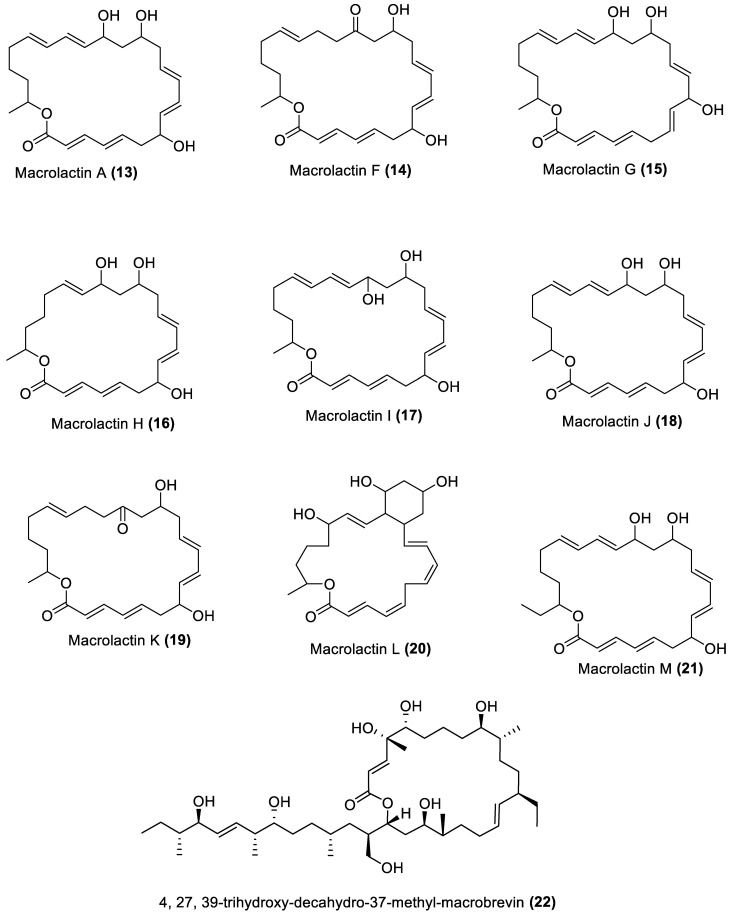

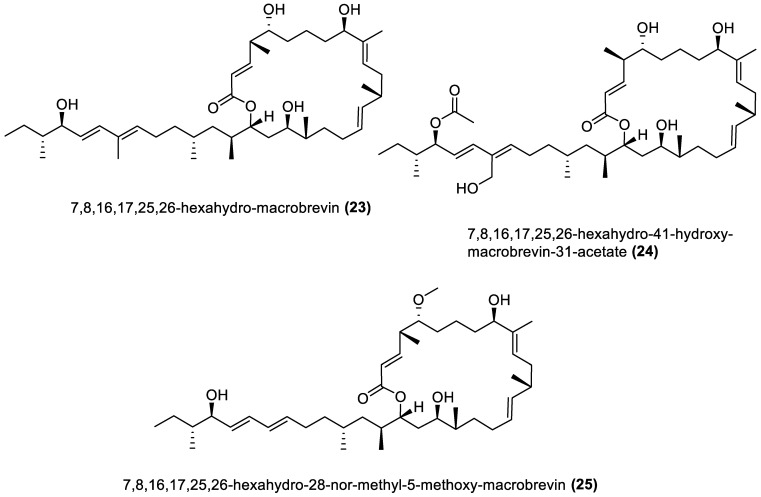
Macrolides from marine macroalgae Bacillota.

**Figure 2:**
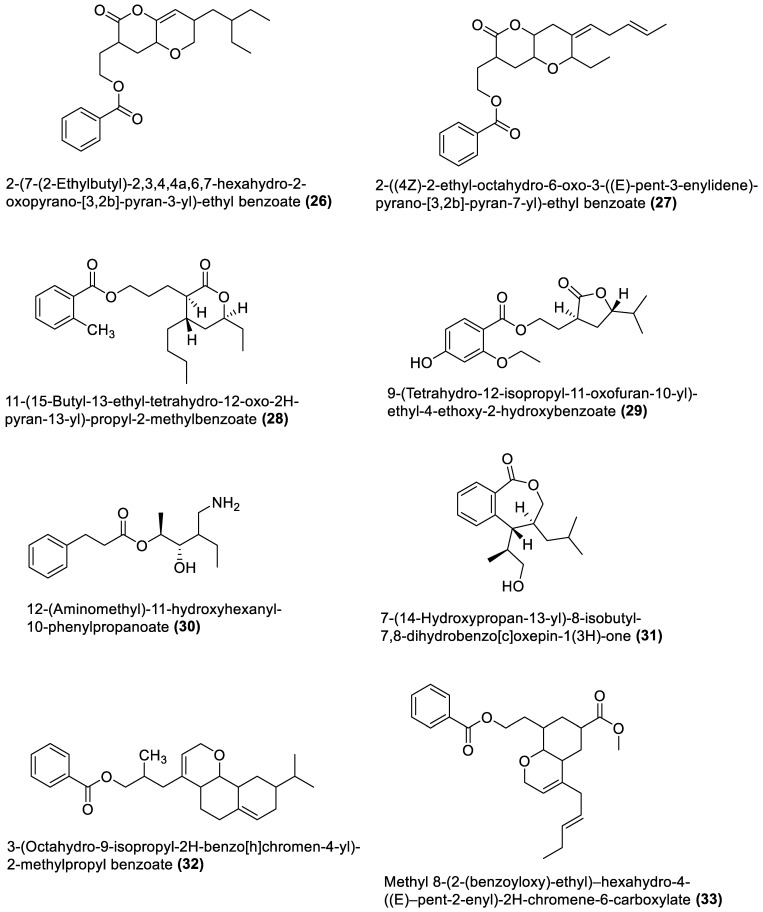

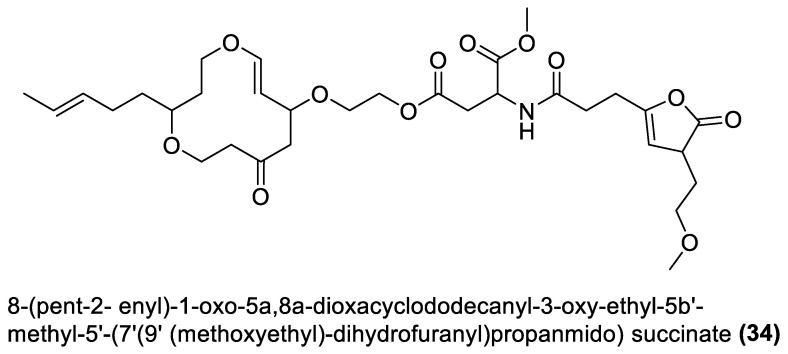
Esters from marine macroalgae Bacillota.

**Figure 3:**
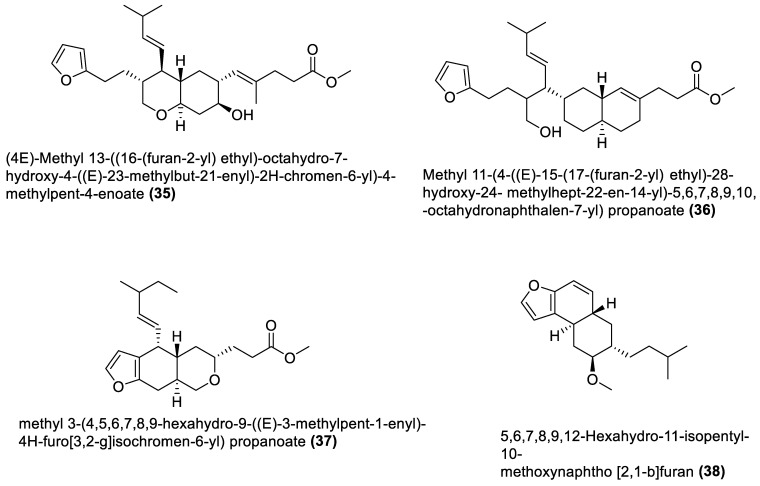
Furanoterpenoids from marine macroalgae Bacillota.

**Figure 4:**
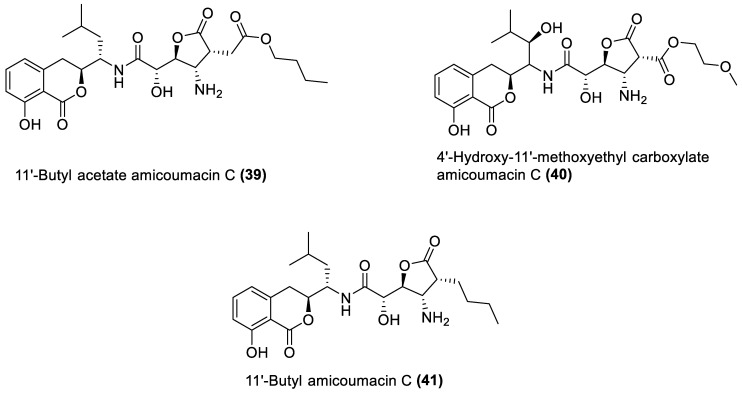
Amicoumacins from marine macroalgae Bacillota.

Table 1 also shows that most bioactive compounds of the marine macroalgae Bacillota belong to the polyketide class, which aligns with the suggestion by Aleti et al., 2015, regarding the prevalence of a polyketide synthase (*pks*) gene cluster in Bacillota [83]. Of the forty-one compounds isolated from the macroalgae Bacillota, thirty-eight are polyketides, while the remaining three belong to the non-ribosomal peptide–polyketide hybrid. The chemical structures of these molecules (**1**–**41**) are shown in Figure 1, Figure 2, Figure 3 and Figure 4, and they are grouped according to their chemical classes, including macrolides, esters, furanoterpenoids and amicoumacins. Apart from the forty-one isolated and characterised compounds, there are forty-seven volatile compounds identified in an extract of a *B. amyloliquefaciens* strain isolated from a brown alga *Zonaria tournefortii* [84], as well as a YbdN protein isolated from *B. licheniformis* of *Fucus serratus* [82].

**Table 1:** Natural products and pharmacological properties of symbiotic Bacillota from marine macroalgae.

Algae Species	Growth Medium	Bacterial Species	Biosynthetic Gene Cluster	Extract/ Compounds	Pharmacological Properties	MIC (µg/mL)	References
**Brown Algae Bacillota**							
*Sargassum wightii*	^a^ ZMA * ^b^ NA ^a^ NA ^a^ ZMA	*Bacillus* species *Bacillus atrophaeus* MW821482	*pks* *pks* *nrps* Siderophore	Ethyl acetate extract Ethyl acetate extract	Antibacterial Antioxidant Antihypertensive Antihypercholesterolamic Anti-inflammatory Anti-hyperglycemic Cytotoxicity Antioxidant Antibacterial Anti-inflammatory Anti-hyperglycemic Antihypertensive Antioxidant Anti-hypercholesterolemic Antibacterial	6.25–12.5 ^⁑^ (133–492.04) ^⁑^ (498.12–735.42) ^⁑^ (10.21–24.32) ^⁑^ (5.22–735.45) ^⁑^ (92.02–759.24) ^♯^ 29.5 ^♯^ (133–4167) 6.25–12.5 ^⁑^ (9.74–788.8) ^⁑^ (118.1–513.4) ^⁑^ 713.6 ^⁑^ (413.2–429.8) ^⁑^ 22.23 6.25–12.5	[85,86,87,88]
*Anthophycus longifolius*	^a^ NA ** NA SWA ZMA * NA MA * NA	*Bacillus subtilis* MTCC 10403	*pks* *pks* *pks*	(**1**) (**35**–**38**) (**2**)	Antibacterial Antibacterial Antibacterial	3.12–50 3.12–25 ND	[89,90,91]
*Sargassum myriocystum*	MA * NA	*Bacillus subtilis* MTCC 10407	*pks*	(**26** and **27**)	Antibacterial	ND	[92]
*Fucus serratus*	^a^ TSA DSTA MA NA * CB	*Bacillus licheniformis*	ND	YbdN protein	Antibacterial	ND	[82]
*Endarachne binghamiae*	MA MB	*Bacillus* sp.	ND	Acetone extract	Antibacterial	188.1–209.7	[93]
*Sargassum muticum*	MA MB	*Bacillus* sp.	ND	Acetone extract	Cytotoxicity Antibacterial	^♯^ 5.5 174	[93]
*Egregia menziesii*	MA MB	*Bacillus* sp.	ND	Acetone extract	Antibacterial	203.0–212.3	[93]
*Padina gymnospora*	^a^ NA ** NA SWA ZMA * NA	*Bacillus amyloliquefaciens*	*pks*	(**28**–**31**)	Antibacterial	ND	[94]
*Zonaria tournefortii*	^d^ LB	*Bacillus amyloliquefaciens* S13	ND	Volatile compounds	Antimicrobial	64–>500	[84]
**Red algae Bacillota**							
*Hypnea valentiae*	^a^ ZMA * MBSA	*Bacillus amyloliquefaciens* MB6 (MTCC 12716)	*pks* *pks-nrps* ND	(**3**–**5**) and (**6**–**8**) (**39**–**41**) Ethyl acetate extract	Antibacterial Antibacterial Antibacterial Anti-inflammatory Anti-hypercholesterolemic Antidiabetic Antioxidant Antibacterial	0.38–5.00 ^¶^ (−9.06)–(−10.13) ^¶^ (−11.33)–(−13.61) 0.78–3.12 3.125–12.5 ^⁑^ (6.06–675.36) ^⁑^ 17.30 ^⁑^ (84.00–639.54) ^⁑^ (136.78–278.19) 6.25–12.50	[95,96,97,98,99,100]
*Kappaphycus alvarezii*	^a^ ZMA * MBSA	*Bacillus amyloliquefaciens* MTCC 12713	*pks* *pks*	(**9**–**12**) (**22**–**25**)	Antibacterial Antibacterial	^‡^ 2–9 × 10^−3^ 1.56–6.25 ^¶^ (−9.06)–(−12.61)	[101,102]
*Laurencia papillosa*	^a^ NA ^c^ ZMA * NA ^a^ ZMA ^a^ NA * NA ^a^ NA ** NA SWA ZMA * NA	*Bacillus velezensis* MBTDLP1 MTCC 13048 *Bacillus velezensis* MBTDLP1 *Bacillus amyloliquefaciens*	*pks* ND *pks*	(**34**) Ethyl acetate extract (**32** and **33**)	Antibacterial Antibacterial Anti-inflammatory Cytotoxicity Antidiabetic Antioxidant Antibacterial	0.38 7.5–15 ^♯^ 17 ^♯^ (32.3–200) ^♯^ (120–420) ^♯^ (107–4127) ND	[103,104,105]
*Laurencia pacifica*	MA MB	*Bacillus* sp.	ND	Acetone extract	Antibacterial	288.1	[93]
*Centroceras clavulatum*	MA MB	*Bacillus* sp.	ND	Acetone extract	Antibacterial	217.1	[93]
*Schizymenia dubyi*	MB	*Bacillus* sp. PP19-H3	*pks*	(**13**–**21**)	Antibacterial	10–>100	[73]
**Green Algae Bacillota**							
*Codium fragile*	MA MB	*Bacillus* sp.	ND	Acetone extract	Antibacterial	196	[93]

LB: Luria–Bertani; MB: marine broth; MA: marine agar; MBSA: modified basal-salt agar; NA: nutrient agar; SWA: seawater agar; ZMA: Zobell marine agar; TSA: tryptone soya agar; DSTA: diagnostic sensitivity test agar; CB: Columbia broth; *pks*: polyketide synthase; *nrps*: non-ribosomal peptide synthetase. MIC: minimum inhibitory concentration; ND: not determined. ^a^ Supplemented with NaCl (1%); ^b^ Supplemented with NaCl (2%); ^c^ Supplemented with NaCl (7%); ^d^ Supplemented with NaCl (15%). ** Half-strength agar; * Fermentation medium. ^‡^ MIC in (µM); ^⁑^ IC_90_ in µg/mL; ^♯^ IC_50_ in µg/mL; ^¶^ Binding energy in kcal/mol.

### 4.1. Macrolides

These are polyketide macrocyclic lactones of varying ring sizes [106]. They are highly oxygenated polyenes [107] with a broad spectrum of antibacterial effects against pathogenic bacteria, which they achieve by binding to the 50S ribosomal subunit of bacteria and disrupting protein synthesis. Erythromycin and azithromycin, with 14- and 15-membered rings, belong to the first- and second-generation macrolide antibiotics [108].

Twenty-five macrolides belonging to different classes have been isolated from marine macroalgae Bacillota, including derivatives of macrolactin (**1**, **2**, **13**–**21**) [73,91], bacvalactone (**3**–**5**), elansolid (**6**–**8**) [95,96,97], difficidin (**9**–**12**) [101], and macrobrevin (**22**–**25**) [102]. Compounds **1** and **2** are 24-membered macrolactins isolated from *B. subtilis* of a brown alga, *Anthophycus longifolius* [89,91]. They are biosynthesised by the type-1 *pks* gene cluster through decarboxylative Claisen condensation, ketoreduction, dehydration and cyclisation reaction steps for **1**. The bacvalactones (**3**–**5**) and elansolids (**6**–**8**), which are also biosynthesised by several decarboxylative Claisen condensation reactions, were isolated from a strain of *B. amyloliquefaciens* of a red alga, *Hypnea valentiae.* The bacvalactones are 24-membered macrolactones with 13-*O*-ethyl (**3**) and 15-*O*-furanyl (**4** and **5**) substituents. The elansolids are 19-membered macrocyclic lactones which contain octahydroisobenzofuran derivative (**6**), 4a,5,7,7a-tetrahydro-2*H*-furo[3,4-b]pyran derivative (**7**) and hexahydro-2*H*-furo[3,4-b]pyran derivative **(8**) in their structure. In addition, 22-membered difficidin (**9**–**12**) and macrobrevin (**22**–**25**) analogues, isolated from *Kappaphycus alvarezii*’s (a red alga) *B. amyloliquefaciens*, are also the products of repeated decarboxylative Claisen condensation reactions of the *pks* gene clusters. Other macrolides of marine algae Bacillota are a set of macrolactins (**13**–**21**) isolated from a red alga (*Schizymenia dubyi*) *Bacillus* sp. All these macrolides were tested only for antibacterial properties, even though they have been reported elsewhere to possess anticancer, neuroprotective, antidiabetic and anti-inflammatory properties [107].

### 4.2. Esters

Different previously undescribed derivatives of heterocyclic (**26**–**29** and **31**–**34**) and aliphatic (**30**) esters have been isolated and characterised from Bacillota of macroalgae (Figure 2). For example, compounds (**26** and **27**), isolated from *B. subtilis* of *Sargassum myriocystum*, are *pks*-1 gene products and members of the pyranyl benzoate analogues [92]. They are synthesised through the Claisen condensation, dehydration and ketoreduction pathways, and resemble two compounds isolated from the alga host. On the other hand, *B. amyloliquefaciens*, isolated from *Padina gymnospora* (a brown alga), produced polyketides (**28**–**31**) through the *pks*-1 gene cluster [94]. The heterocyclic esters (**32** and **33**), which are octahyrobenzopyran derivatives and the secondary metabolites of *B. amyloliquefaciens*, isolated from a red alga, *Laurencia papillosa*, are also *pks* gene products [105]. In addition, the macrocyclic diester (**34**), isolated from *B. velezensis* of *Laurencia papillosa* (a red alga), also belongs to the *pks*-1 gene products [103]. Like the macrolides, these nine esters were only tested for antibacterial properties.

### 4.3. Furanoterpenoids

Furanoterpenoids are a class of terpenoids containing at least a furan ring [109]. Many furan-containing compounds, including furanoterpenoids, are toxic to humans. Their toxicity is through the cytochrome P450-catalysed oxidation of the furan ring to two reactive electrophilic intermediates that can bond with macromolecules and cause toxicity [110,111]. However, furanoterpenoids have been reported to elicit anti-inflammatory [112], antimalarial [113], and other properties [111]. Furanoterpenoids (**35**–**38**) isolated from *B. subtilis* of red alga (*Anthophycus longifolius*) showed in vitro antibacterial effects [90]. Two were sesterpenoid-type compounds (**35**, **36**), and the others were furan annulation compounds. However, considering the toxicity concern regarding this group of compounds, an in vivo toxicity assay of any furan-containing molecule would be necessary, using an animal model, to ascertain their safety. The four furanoterpenoids are biosynthesised by the *pks* gene cluster.

### 4.4. Amicoumacin C Derivatives

Amicoumacins are derivatives of the dihydroisocoumarin class of compounds biosynthesised by bacterial non-ribosomal peptide–polyketide (nrp-pk) hybrid biosynthetic pathway [114]. Out of the forty-one natural products of the macroalgae Bacillota, the amicoumacins (**39**–**41**) are the only chemical compounds produced by another gene cluster other than the *pks*, even though non-ribosomal peptides synthetase (*nrps*) gene clusters are quite common in *Bacillus* species [101,115]. There is, therefore, a need to explore different biosynthetic pathways of macroalgae Bacillota for diverse bioactive chemical species. The structures of **39**–**41** are shown in Figure 4, and they were isolated from the *B. amyloliquefaciens* of a red alga, *Hypnea valentiae* [98]. Although amicoumacins have been reported to possess varying biological effects such as antibacterial, anti-ulcer, anti-inflammatory and cytotoxic effects [116], in our survey of amicoumacins produced by macroalgae Bacillota, only antibacterial effects were reported.

## 5. Pharmacological Properties of the Secondary Metabolites of Marine Macroalgae Bacillota

In addition to the chemical constituents of the marine macroalgae Bacillota, Table 1 also captures the biological activities of the compounds numbered **1**–**41** and the extracts. Among the marine Bacillota, *Bacillus* species are the most common symbionts of macroalgae, and they often showcase higher antibacterial properties than their counterparts [117]. Furthermore, species of *Bacillus* dedicate more than 7% of their genomes to producing compounds with antimicrobial properties [118]. These two assertions can be seen clearly in Table 1: *Bacillus* were the only species isolated from all the macroalgae included in the table for bioactive natural products, and mainly elicited antibacterial properties.

During this survey, we realised that some authors carried out preliminary bioassays only, using bacterial cultures instead of extracts or isolated chemical compounds from the bacteria [81,117,119,120,121,122,123,124,125,126,127,128,129]. Another scenario is where the antimicrobial effects of bacterial extracts/fractions were checked without determining the basic bioassay parameters like MIC, IC_50_ or GI_50_ [130,131,132,133,134,135]. The studies mentioned above were omitted in Table 1. However, the table captures chemical constituents with low therapeutic properties—MIC/IC_50_/GI_50_ values greater than 100 and 10 µg/mL—for crude extracts and pure compounds, respectively, and compounds whose basic bioassay parameters were not determined. Though they might not be suitable hits/leads for the reported activities [136,137], we included them in the table because those chemical constituents might have other viable biological properties if assessed.

### 5.1. Antibacterial Property of Marine Macroalgae Bacillota

Mostly in vitro antibacterial properties were reported for macroalgae Bacillota, according to data in Table 1. The high frequency of documented antibacterial properties might be due to the ease of carrying out this assay in the laboratory, as opposed to other bioassays. Another potential explanation could be the author’s intention to demonstrate the antifouling properties attributed to symbiotic bacteria associated with macroalgae. The compounds isolated from the macroalgae Bacillota showed varied levels of antibacterial effects against human bacterial pathogens. The best in vitro antibacterial activity (with the MIC value of 2–9 × 10^−3^ µM) was exhibited by the difficidin analogues (**9**–**12**) isolated from *B. amyloliquefaciens* associated with a red alga, *Kappaphycus alvarezii* [101]. These compounds showed bactericidal activities against a broad spectrum of pathogenic bacteria, including methicillin-resistant *Staphylococcus aureus* (MRSA) and vancomycin-resistant *Enterococcus faecalis* [101]. Other compounds with appreciable antibacterial activity are **7** and **34**. At an MIC value of 0.38 µg/mL, compound **7**, a product of *B. amyloliquefaciens* isolated from a red alga, *Hypnea valentiae*, was active against MRSA and *Vibrio haemolyticus* [96,97], similar to **34**, produced by *B. velezensis* of *Laurencia papillosa* [103]. Worthy of mention is **40** (MIC value: 0.78 µg/mL), another active compound from *B. amyloliquefaciens* of *Hypnea valentiae*, with a broad spectrum activity against pathogenic bacteria [98]. Table 1 lists the MIC values of other compounds and extracts of macroalgae Bacillota. Unfortunately, as seen from the table, some extracts with MIC values lower than 100 µg/mL were not further simplified to isolate their bioactive chemical constituents.

### 5.2. Other Pharmacological Properties of Marine Algae Bacillota

Other biological properties, such as cytotoxicity, anti-inflammatory, antioxidant, antidiabetic, anti-hypercholesterolemic and anti-hyperglycemic, were exclusively determined for extracts/fractions of the macroalgae Bacillota. For example, the only reported in vitro antifungal assay was recorded for a volatile fraction of *B. amyloliquefaciens* isolated from *Zonaria tournefortii* [84]. Another example of biological activities of the macroalgae Bacillota extract is that of an acetone extract of a *Bacillus* species isolated from a brown alga, *Sargassum muticum*. The acetone extract displayed a good in vitro cytotoxic effect (IC_50_ value of 5.5 µg/mL) against colon cancer cells [93], but it was not purified further to pure compounds. The concentrations of other extracts of the macroalgae Bacillota with in vitro biological effects are given in Table 1.

## 6. Conclusions

Bacillota from marine macroalgae are an excellent source of potent, novel chemical compounds. However, it is evident from this overview that more macroalgae should be investigated for chemical constituents of their Bacillota, as only a fraction of over nine thousand macroalgae [138] have been covered. In addition, only in vitro and a few in silico antibacterial assays were carried out for chemical compounds of the studied macroalgae Bacillota, which is insufficient for a drug discovery process. It would, therefore, be appropriate to accompany the in vitro/in silico assays with in vivo bioassays to establish the safety and bioavailability of those potent antibacterial molecules. In another vein, carrying out only antibacterial assays for new natural products is quite limiting when other health problems (like cancer) are also a concern. Therefore, we suggest more diverse biological assays for the isolated molecules of macroalgae Bacillota, to expand their bioactivity profiles.

## Data Availability

Not applicable.
